# How prone are Swedish general practitioners to perform medication reconciliation? A theory-based survey study

**DOI:** 10.1177/20420986251360916

**Published:** 2025-07-25

**Authors:** Sarah Thelin, Sara Modig, Veronica Milos Nymberg

**Affiliations:** Center for Primary Health Care Research, Department of Clinical Sciences Malmö, Lund University, Jan Waldenströms gata 35, Malmö 21428, Sweden; University Clinic Primary Care, Skåne University Hospital, Region Skåne, Sweden; Center for Primary Health Care Research, Department of Clinical Sciences Malmö, Lund University, Malmö, Sweden; University Clinic Primary Care, Skåne University Hospital, Region Skåne, Sweden; Department of Medicines Management and Informatics in Skåne County, Malmö, Sweden; Center for Primary Health Care Research, Department of Clinical Sciences Malmö, Lund University, Malmö, Sweden; University Clinic Primary Care, Skåne University Hospital, Region Skåne, Sweden

**Keywords:** adverse drug events, drug-related problems, medication reconciliation, physician, primary care, the theory of planned behaviour

## Abstract

**Background:**

Drug-related problems are common in older individuals. A medication reconciliation has the goal of identifying and maintaining an accurate medication list and can serve to prevent drug-related problems caused by discrepancies.

**Objectives:**

This study aimed to explore primary care physicians’ intentions towards performing medication reconciliation in patients with multimorbidity using a theory-based questionnaire.

**Design:**

A survey study was conducted from February to March 2024.

**Methods:**

An anonymous web-based questionnaire was developed, validated and distributed to 674 primary care physicians in southern Sweden. The questionnaire targeted attitudes, perceived norms, perceived behavioural control and generalised intentions towards performing a medication reconciliation, constructs derived from the theory of planned behaviour and the reasoned action approach theory. Outcome measures were overall scores for predictors, and the correlation between predictors and intentions towards performing a medication reconciliation was analysed using a multiple linear regression model.

**Results:**

With 206 surveys answered, the response rate was 31%. We found items targeting attitudes to have the highest overall mean score on a seven-point Likert scale (6.42), followed by generalised intention (6.17), subjective norms (5.45) and perceived behavioural control (5.15). Women had significantly higher scores for attitudes (*p*-value 0.001), subjective norms (*p*-value 0.050) and generalised intention (*p*-value 0.001). Groups with more than 10 years of work experience had significantly higher overall mean scores for perceived behavioural control (*p*-value 0.043). The correlation between predictors and generalised intention found attitudes and perceived behavioural control to be significant predictors of intentions to perform medication reconciliation in multimorbid older individuals (*p*-value < 0.001).

**Conclusion:**

We found attitudes and perceived behavioural control to be significant predictors of primary care physicians’ intention to perform a medication reconciliation in patients with multimorbidity. These findings provide important insights into how future interventions targeting behavioural predictors can be developed.

## Plain language summary

How likely are Swedish general practitioners to update patients’ medication lists?

Why was the study done?

Older individuals are susceptible to drug-related problems which can be caused by medication lists not being accurate. Medication reconciliation is a process where physicians check and update a patient’s list of medications to avoid errors that could harm the patient. This study aimed to understand why primary care physicians in Sweden might or might not perform medication reconciliations for older patients with multiple health conditions.

What did the researchers do?

The researchers used a theory called the ‘Theory of Planned Behaviour’ to design a survey, focusing on the physicians’ attitudes, social influences, and perceived ability to perform this task. The survey was sent by e-mail to 674 physicians in southern Sweden, with 206 responses.

What did the researchers find?

The results showed that physicians’ attitudes (how they feel about the benefits of medication reconciliation) and their perceived control over the task were the most significant factors predicting whether they intended to perform medication reconciliations. Female physicians and those with more than 10 years of professional experience were more likely to have positive attitudes and feel confident in performing this task.

What do the findings mean?

The study concluded that future efforts to improve medication reconciliation should focus on changing physicians’ attitudes and increasing their confidence in handling this task. This could help prevent medication-related issues in older patients, who are particularly vulnerable to drug-related problems due to their complex health needs.

## Introduction

Drug-related problems (DRPs) are defined as ‘an event or circumstance involving drug therapy that actually or potentially interferes with desired health outcomes’.^1^ These preventable negative health outcomes are frequently seen in older individuals.^2^ Inappropriate or high-risk prescribing, as well as medication discrepancies leading to DRPs, has been targeted with different methods such as medication reviews,^3^ medication reconciliations^4^ or educational staff interventions.^5^ Evidence of such interventions has shown varied effects on hospitalisations, mortality or quality of life.^6^ Medication discrepancies, with inconsistencies between the medication lists at different healthcare providers, pharmacies and the medication used by the patient, are often unintended but have the potential to cause harm by addition, omission or change in dosage.^7^

A recent Swedish study showed that 72% of the medication lists in the electronic medical records for patients in both primary and secondary care contained at least one medication discrepancy in a point prevalence measurement.^8^ Different factors might contribute to medication discrepancies: the variety of electronic medical records at different care providers^9^ and poor prescription or poor adherence.^10^ Furthermore, a review of DRPs after transitioning from hospital to the home found medication discrepancies and inadequate medication reconciliation to be accountable for 40% of DRPs, with studies reporting medication discrepancies found in 56% and 94% of older people, respectively.^11^

**Figure 1.**
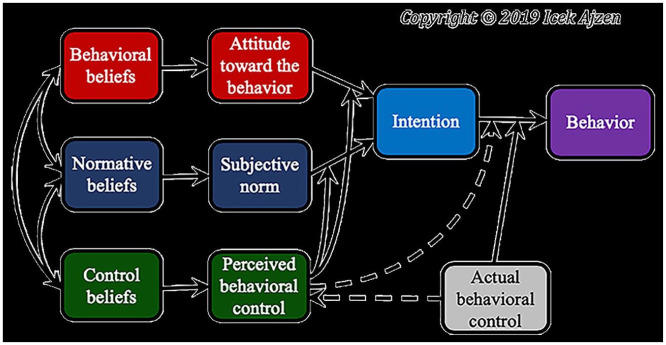
Schematic representation of the theory of planned behaviour.^21^

A medication reconciliation involves revising a person’s complete list of medications, by checking for accuracy, compliance and documenting any changes.^4^ In primary care, medication reconciliations are conducted during patient assessments, when prescribing medication, and for annual health reviews, using sources like patient interviews, electronic health records, pharmacy records and previous medication lists. Unlike a medication review, which focuses on optimising a patient’s drug treatment regarding age, kidney function and potential DRPs, a medication reconciliation has the goal of identifying any discrepancies and maintaining an accurate and updated medication list.^12^ This is important during transitions in care^13^ when medication discrepancies often occur.^14,15^ Even if there is evidence showing that medication reconciliation might prevent DRPs, there are few studies demonstrating how this might be implemented effectively in a primary care setting.^16,17^ As several factors might influence physicians’ decision-making, understanding and modifying clinicians’ behaviour has been described from a theoretical point of view.^18^ One of the theories proposed to explain clinicians’ prescribing behaviour is the theory of planned behaviour (TPB).^19,20^ The theory proposes that attitudes, social norms and perceived behavioural control are predictors of behavioural intention, which, in turn, is a strong predictor of behaviour (Figure 1).^20^

**Figure 2.**
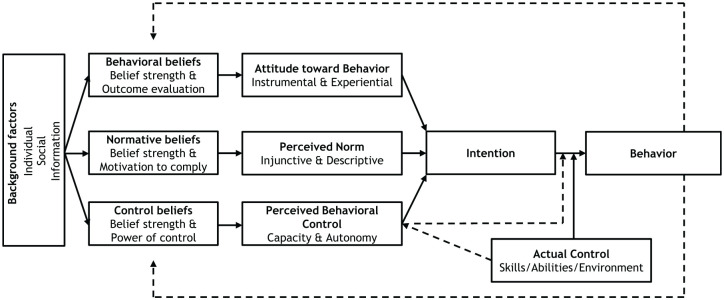
Graphical representation of the reasoned-action approach.^28^

According to the theory, attitude towards a behaviour has two components: beliefs about the consequences of the behaviour and the corresponding positive or negative judgements about the behaviour. Subjective norms are the individual’s own perceived social pressure to perform or not perform a certain behaviour. Perceived behavioural control is the extent to which the individual feels able to perform a behaviour, both in terms of self-efficacy and control beliefs about the power to influence situational or internal factors to inhibit or facilitate a behavior.^22^ TPB is therefore a useful theoretical framework to describe, predict and even alter behaviours. For instance, TPB-based questionnaires have been used to assess, predict and influence primary care physicians’ behaviour in antibiotic prescribing,^23^ or their intention to use electronic learning tools.^24^ In recent years, researchers have described a further development of TPB, called the reasoned action approach (RAA), by differentiating subcomponents of the model^25^ (Figure 2). RAA has been used to study the predictive value of attitudes and intentions in physicians’ prescribing behaviour.^26,27^ The theory has been found to explain 58.7% and 32.3% of the variance in intention and behaviour, respectively, which is considerably higher than previously reported values for intention (44.3%) and behaviour (19.3%).^25^

We found no previous survey study based on cognitive behavioural theories assessing physicians’ behavioural intentions to make a reconciliation of patients’ medication lists in patients with multimorbidity and polypharmacy.

### Aim

The first aim of this study was to validate a theory-based questionnaire targeting attitudes, perceived norms, perceived behavioural control and generalised intentions towards performing a medication reconciliation in patients with multimorbidity.

The second aim was to describe the theoretical constructs derived from the TPB and RAA in primary care physicians and to analyse the association between predictors of behavioural intention (attitudes, perceived norms and perceived behavioural control) and the behavioural intention to perform a medication reconciliation, with the intent of using relevant predictors in future efforts to increase medication reconciliations.

## Methods

### Study design and population

This survey study was conducted from February to March 2024. An anonymous web-based questionnaire was sent to a total of 674 primary care physicians in Skåne County in southern Sweden.

### Construction of the survey

The questionnaire was designed using a manual based on the TPB.^29^ The questions were targeted to assess expected predictors of prescribing behaviour, such as past experiences regarding the respondent’s current strategy in performing a medication reconciliation, attitudes towards potential benefits as well as perceived norms and perceived control and finally, behavioural intention (see Supplemental File 1). Answers were reported on a seven-point Likert scale^30^ ranging from strongly disagree to strongly agree.

Construct validity and readability of the survey in paper format were assessed by two physicians to ensure the relevance of items and wordings, and led to minor changes in wording and formatting. The survey was then pilot-tested at three primary health care centres by distributing the questionnaire to 20 primary care physicians. For each of the primary outcome variables (attitudes, perceived norms, perceived behavioural control and generalised intention), the mean of the item scores was calculated to give an overall score. Missing values were replaced with the mean value for the item (imputation).

Internal consistency was measured with Cronbach’s α coefficient (CA) with an accepted value >0.65, with each item being validated independently. For items with low CA, subitems were removed to improve CA. None of the main items were removed, and the reduction resulted in fewer subitems concerning all four predictors. For instance, the first item targeting attitudes had originally eight subitems; however, after removing subitems with low reliability, five subitems remained. In total, 42 subitems were reduced to 29. A lower CA was noted amongst questions with an inverted scale, thus resulting in alterations to ensure a unanimous scale throughout the survey, without the use of an inverted scale.

Temporal stability of the questionnaire was assessed by redistributing the questionnaire to the same 20 respondents after 2 weeks. For identifying individual respondents, whilst they remained anonymous, they were asked to mark their survey sheets with a personal code or figure. The Pearson correlation coefficient was calculated to examine test–retest reliability. This led to a change in the structure of some questions to improve the final questionnaire. Table 1 shows the items of the different predictors.

**Table 1.** Items targeting each predictor in a TPB-based questionnaire evaluating physicians’ intention to perform medication reconciliation.

Attitudes	In general, to update the medication list: (a) Reduces the risk of unnecessary prescribing (b) Reduces the risk of admissions (c) Increases patient safety (d) Reduces the time required for the entire doctor’s visit in the long term (e) Reduces the likelihood of drug-related problems
	How important is it to. . . (a) avoid prescribing unnecessary drugs? (b) reduce the risk of drug-related hospitalisations? (c) reduce the likelihood that the patient will seek treatment again due to a drug-related problem? (d) have enough time for medication reconciliation during the patient’s visit? (e) reduce the patient’s risk of drug-related problems in the future?
	If I routinely manage multimorbid patients by updating the medication list, my life as a general practitioner will generally be easier in the long run
	Treating multimorbid patients by updating the medication list is. . . (a) usually a better treatment option (b) satisfactory more often than unsatisfactory
Subjective norms	About updating the medication list: (a) Many people who are important to me (colleagues, patients, the manager) think that I should update medication lists for multimorbid patients (b) I am expected to update medication lists for multimorbid patients
	When it comes to updating a patient’s medication list, how motivated are you to do what. . . (a) the primary care colleagues think you should (b) the inpatient colleagues think you should (c) the manager thinks you should
Perceived behavioural control	How confident are you in your ability/competence (a) to update the medication list at each visit for multimorbid patients? (b) to end a visit for a multimorbid patient whom you have treated by handing out the medication list?
	With the current working methods and conditions, how confident are you in your own ability to update the medication list in patients with multimorbidity who (a) come in for a scheduled check? (b) have multiple prescribers? (c) use many medications?
	Based on the information you have from the visit: (a) I want to and can update the medication list in patients with multimorbidity. (b) I am convinced that I can update the medication list in multimorbid patients, even with several prescribers (c) I can overcome all obstacles (e.g. lack of time), to update the medication list in patients with multimorbidity
Generalised intention	Management of patients with multimorbidity. (a) When managing multimorbid patients, I automatically plan to update their medication list. (b) I want to treat multimorbid patients by updating their medication list. (c) I strive to manage multimorbid patients by updating their medication list.

TPB, theory of planned behaviour.

### Collection of data

The questionnaire was distributed to primary care physicians in southern Sweden by e-mailing an invitation with an individual link using the electronic database manager Sunet Survey. The invitation e-mail outlined the purpose of the study, the approximate length of time the survey would take to complete, contact information of the principal investigator for questions and the anonymous nature of their participation, as well as containing an individual link to the questionnaire. Two additional e-mails with reminders were sent to non-responders. Upon answering the survey, their response was deidentified and coded automatically by the software. To ensure good generalisability and response rate, we targeted all practicing primary care physicians in Skåne County using an updated e-mail list usually used for educational purposes (meetings and guideline dissemination).

### Outcome measures

Primary outcomes were overall scores from items targeting the predictors attitudes, subjective norms, perceived behavioural control and generalised intention to perform a medication reconciliation in patients with multimorbidity. Secondary outcomes were the correlation between generalised intention and attitudes, subjective norms and perceived behavioural control, adjusted for sex, age, work experience and past behaviour.

### Analysis

The primary outcome variables were calculated to give an overall score, presented with median due to the non-normally distributed scores. Group comparison analyses were performed using Mann–Whitney test and Kruskal–Wallis test. Spearman’s rank order correlation was used to study associations between the different items. The correlation between predictors and intentions was assessed using multiple linear regression models. Data were analysed using IBM SPSS Statistics v29,^31^ with statistical significance at the 5% level. The CHERRIES checklist was followed for reporting results of internet e-surveys^32^ (see Supplemental File 2).

### Ethics

All participants who completed the questionnaire provided informed consent to participate in the study. No patient data were collected in this study, and an advisory statement was received from the Swedish Ethical Review Authority (Registry number 2023-01831-01) confirming that the study is not covered by the regulations in §§ 3–4 of the Ethics Review Act and must therefore not be ethically reviewed.

## Results

A total of 206 surveys were answered, with a response rate of 31%. Characteristics of the respondents are outlined in Table 2. For items targeting the same predictor, the overall mean score on a seven-point Likert scale was highest for attitudes, followed by generalised intention, subjective norms and perceived behavioural control (Table 2). Results for individual variables are available (see Supplemental File 3).

**Table 2.** Characteristics of study participants.

Variable	
Sex, % (*n*)	
Women	56% (115)
Men	44% (91)
Age, % (*n*)	
<35 years	35% (73)
36–45 years	35% (71)
46–55 years	18% (37)
56–65 years	9% (19)
>65 years	3% (6)
Work experience, % (*n*)	
0–3 years	15% (32)
4–10 years	51% (105)
11–20 years	19% (40)
>21 years	14% (29)
Past behaviour, median (IQR)	
Medication reconciliations performed on multimorbid patients, out of the 10 latest patient visits (self-reported)	8 (3)
Current working method includes performing a medication reconciliation on multimorbid patients (self-reported), on a seven-point Likert Scale*	6 (1)
Overall mean score for items targeting the same predictor on a seven-point Likert scale*, median (IQR)	
Attitudes	6.54 (0.6)
Subjective norms	5.60 (1.8)
Perceived behavioural control	5.25 (1.4)
Generalised intention	6.33 (1.3)

* 1 = strongly disagree, 7 = strongly agree.

IQR, interquartile range.

The internal consistency was confirmed with a CA between 0.67 and 0.86. An exploratory factor analysis with Varimax rotation and principal component analysis showed a Keiser–Meyer–Olkin value of 0.88 (*p* < 0.001) and resulted in seven-factor components with an Eigen value of 1.03–8.84 cumulatively explaining 67.3% of the variance (Table 3).

**Table 3.** Exploratory factor analysis with varimax rotation and principal component analysis.

Items in factor	Factor						
	1	2	3	4	5	6	7
13b. With the current working methods and conditions, how confident are you in your ability to update the medication list in patients with multimorbidity who have multiple prescribers?	0.85						
13c. With the current working methods and conditions, how confident are you in your ability to update the medication list in patients with multimorbidity who use many medications?	0.82						
14b. Based on the information you have from the visit, I am convinced that I can update the medication list in multimorbid patients even with several prescribers	0.77						
14c. Based on the information you have from the visit, I can overcome all obstacles (e.g. lack of time) to update the medication list in patients with multimorbidity	0.75						
13a. With the current working methods and conditions, how confident are you in your ability to update the medication list in patients with multimorbidity who come in for a scheduled check?	0.71	0.36					
14a. Based on the information you have from the visit, I want to and can update the medication list in patients with multimorbidity.	0.70						
15a. Management of patients with multimorbidity. When managing multi-morbid patients, I automatically plan to update their medication list.	0.56			0.41		0.33	
Eigenvalue Factor 1	8.84						
Variance explained by Factor 1	30.5%						
7c. How important is it to. . . reduce the likelihood that the patient will seek treatment again due to a drug-related problem?		0.78					
7b. How important is it to. . . reduce the risk of drug-related hospitalisations?		0.68	0.32				
7e. How important is it to. . . reduce the patient’s risk of drug-related problems in the future?		0.65					0.34
7a. How important is it to. . . avoid prescribing unnecessary drugs?		0.64					
12b. How confident are you in your ability/competence to end a visit for a multimorbid patient whom you have treated by handing out the medication list?	0.38	0.56					
12a. How confident are you in your ability/competence to update the medication list at each visit for multimorbid patients?	0.35	0.55					
Eigenvalue Factor 2		3.16					
Variance explained by Factor 2		10.9%					
6a. In general, to update the medication list, reduces the risk of unnecessary prescribing			0.85				
6e. In general, to update the medication list, reduces the likelihood of drug-related problems			0.75				
6c. In general, to update the medication list: Increases patient safety			0.75	0.36			
6b. In general, to update the medication list, reduces the risk of admissions			0.69				
6d. In general, to update the medication list, reduces the time required for the entire doctor’s visit in the long term			0.52				
Eigenvalue Factor 3			2.30				
Variance explained by Factor 3			7.9%				
9a. Treating multimorbid patients by updating the medication list is. . . usually a better treatment option				0.66			
9b. Treating multimorbid patients by updating the medication list is. . . satisfactory more often than unsatisfactory				0.66			
8. If I routinely manage multimorbid patients by updating the medication list, my life as a general practitioner will generally be easier in the long run			0.32	0.62			
15b. Management of patients with multimorbidity. I want to treat multimorbid patients by updating their medication list.		0.38		0.61		0.33	
15c. Management of patients with multimorbidity. I strive to manage multimorbid patients by updating their medication list.		0.35		0.61		0.34	
Eigenvalue Factor 4				1.71			
Variance explained by Factor 4				5.9%			
11c. When it comes to updating a patient’s medication list, how motivated are you to do what. . . the manager thinks you should					0.84		
11b. When it comes to updating a patient’s medication list, how motivated are you to do what. . . the inpatient colleagues think you should					0.84		
11a. When it comes to updating a patient’s medication list, how motivated are you to do what. . . the primary care colleagues think you should					0.82		
Eigenvalue Factor 5					1.36		
Variance explained by Factor 5					4.7%		
10a. About updating the medication list: Many people who are important to me (colleagues, patients, the manager) think that I should update medication lists for multimorbid patients						0.78	
10b. About updating the medication list: I am expected to update medication lists for multimorbid patients					0.34	0.68	
Eigenvalue Factor 6						1.12	
Variance explained by Factor 6						3.9%	
7d. How important is it to. . . have enough time for medication reconciliation during the patient’s visit?							0.84
Eigenvalue Factor 7							1.03
Variance explained by Factor 7							3.6%

Group comparisons were made for sex and work experience. Women had significantly higher scores for attitudes (*p*-value 0.001), subjective norms (*p*-value 0.050) and generalised intention (*p*-value 0.001; Table 4).

**Table 4.** Distribution of overall mean score for items targeting the same predictor on a seven-point Likert scale by sex, median.

Variable	Women	Men	*p* Value*
Attitudes	6.69	6.46	0.001
Subjective norms	5.80	5.20	0.050
Perceived behavioural control	5.38	5.13	0.664
Generalised intention	6.67	6.00	0.001

* Mann–Whitney.

When comparing work experience, a higher mean rank was seen for groups with 11–20 years of work experience and 21 years and more for the predictor perceived behavioural control (*p*-value 0.043; Table 5).

**Table 5.** Distribution of overall mean rank score for items targeting the same predictor on a seven-point Likert scale by work experience, median.

Variable	0–3 years	4–10 years	11–20 years	⩾21 years	*p* Value*
Attitudes	6.77	6.62	6.46	6.54	0.495
Subjective norms	5.80	5.80	5.40	5.80	0.413
Perceived behavioural control	4.88	5.00	5.63	5.50	0.043
Generalised intention	6.33	6.33	7.00	6.33	0.127

* Kruskal–Wallis.

Analysing the effect of the predictors on generalised intention using multiple linear regression, we found attitudes and perceived behavioural control to be significant predictors (*p*-value < 0.001; Table 6). Assumptions for regression analysis were checked. Scatterplots indicated a linear relationship between the predictors and the dependent variable. The Durbin–Watson test indicated no significant autocorrelation, suggesting that the residuals are independent. Homoscedasticity was assessed by plotting the residuals against the predicted values. The plot showed no clear pattern, indicating that the variance of the residuals is constant across levels of the predictors. The normality of residuals test using Q–Q plots showed that the residuals were approximately normally distributed.

**Table 6.** Multiple linear regression for predictors effect on behavioural intention, beta (95% confidence interval, *p*-value).

Predictor	Unadjusted	Adjusted for sex	Adjusted for sex and age	Adjusted for sex, age and work experience	Adjusted for sex, age, work experience and past behaviour
R-squared	0.470	0.482	0.484	0.487	0.545
Attitudes	0.560 (0.376 to 0.745, <0.001)*	0.512 (0.325 to 0.700, <0.001)*	0.515 (0.327 to 0.703, <0.001)*	0.530 (0.341 to 0.720, <0.001)*	0.491 (0.311-0.671, <0.001)*
Subjective norms	0.067 (−0.022 to 0.157, 0.139)	0.058 (−0.031 to 0.148, 0.197)	0.057 (−0.032 to 0.146, 0.208)	0.061 (−0.028 to 1.151, 0.179)	0.047 (−0.038 to 0.132, 0.274)
Perceived behavioural control	0.407 (0.297 to 0.516, <0.001)*	0.416 (0.307 to 0.524, <0.001)*	0.408 (0.297 to 0.518, <0.001)*	0.399 (0.288 to 0.511, <0.001)*	0.216 (0.089–0.344, <0.001)*

* *p*-value < 0.05.

We have calculated the effect sizes for our predictors using Cohen’s formula (f^2^ = \frac{R^2^}{1 − R^2^}), where (R^2^) is the coefficient of determination from our regression analysis. According to Cohen’s guidelines, we interpreted the effect sizes as large, ranging from 0.88 to 1.20.

## Discussion

### Principal findings

In this study, we developed and validated a theory-based questionnaire targeting attitudes, perceived norms, perceived behavioural control and generalised intentions amongst primary care physicians towards performing a medication reconciliation in patients with multimorbidity. We found items targeting attitudes towards performing a medication reconciliation to have the highest overall mean score on a seven-point Likert scale. Significantly higher measures were seen for women for attitudes, subjective norms and generalised intention towards performing a medication reconciliation, suggesting that women are more susceptible to influence regarding attitudes and subjective norms and that women are more likely to perform medication reconciliations. Furthermore, our findings showed that more than 10 years of work experience was associated with higher perceived behavioural control towards performing a medication reconciliation. We found attitudes and perceived behavioural control to be statistically significant predictors of generalised intention towards performing a medication reconciliation in both unadjusted and adjusted models. Further studies are needed to expand upon our findings.

### Comparison with other studies

Previous studies have shown that general practitioners’ perception of their responsibility for their patients’ medication lists varies.^33^ This may result in deficiently updated medication lists, which affect medication safety, as previously identified as a substantial shortcoming in Swedish primary care.^34,35^ Methods for securing an accurate medication list were identified as an urgent improvement need.

In line with our findings, an Irish survey study reported that 98% of general practitioners found medication reconciliation to be an important way to improve medication safety.^36^ Despite this, no formal systems for medication reconciliation were in place in most general practitioners’ practices. Similarly, a Swedish study found that when primary care physicians receive a discharge summary after hospital admission with information on a changed medication list, only one-third of physicians updated the medication list in the primary care medical records.^37^ Regardless of this, two-thirds of the physicians did in fact indicate that they always or often updated the medication list. As such, actual behaviour among physicians does not seem to coincide with self-reported intentions towards performing a medication reconciliation. Similar to both studies is the self-reported nature of the intention towards performing a medication reconciliation. The findings in this theory-based study might provide an increased understanding of how attitudes correlate with behaviour.

We found a significant difference with higher mean rank scores for perceived behavioural control for groups with work experience exceeding 10 years. This finding suggests that substantial experience in the profession leads to a greater sense of behavioural control. This could indicate the need for increased education early on in primary care physicians’ careers on how to perform medication reconciliations. A systematic review of education initiatives aimed at improving trainee skills and knowledge in performing medication reconciliations found early education of medical students to be effective in an intervention setting.^38^ The study also stressed that medical residents should be involved in the development of quality improvement programs. However, only three studies involving resident doctors were identified, which demonstrates the lack of initiative on the matter.

### Strengths and limitations

This is, to our knowledge, the first study to assess the intention to perform a medication reconciliation among primary care physicians based on the TPB, and this is a strength of the study. Furthermore, the study’s design holds several strengths. For instance, digital entry was only available on a single occasion, preventing multiple-entry responders. Also, only fully answered questionnaires were permitted for submission, and a non-response option was not provided, an approach that reduces selection bias by ensuring that all respondents provide complete data. The questionnaire was pilot-tested and validated with CA > 0.6, indicating acceptable internal consistency. However, limitations were also evident. First, the data were self-reported, introducing the risk of recall bias regarding items concerning past behaviour. As in most survey studies, there is a risk of selection bias, with people having stronger opinions being more prone to respond to the survey. Also, the lack of a non-response option might discourage participation and lead to a lower completion rate. The low response rate (31%) is a major limitation in this study; however, it is acceptable compared with other studies on primary care physicians using web-based questionnaires based on TPB, with response rates of 7%–28%.^39–42^ Furthermore, the low response rate may have introduced selection bias, as those who responded to the survey might have different experiences and attitudes from those who did not. This could affect the generalisability of the findings. Also, the reliance on self-reported past behaviour for items like the number of reconciliations performed could introduce inaccuracies with a risk of recall bias. Moreover, there is a risk of response bias due to respondents acknowledging the desirability of certain behaviours and subconsciously inflating their engagement. Likewise, the risk of observer bias is evident by the phrasing of questions to recognise positive behaviours. Furthermore, we did not conduct a formal power calculation prior to the data collection. However, we conducted post hoc power analyses to ensure that our sample size was adequate for detecting the expected effects. These analyses confirmed that our sample size was sufficient to achieve a power of approximately 0.80 and showed an effect size of >0.5, which is generally considered a large effect size. Furthermore, even if we tried to reach as many physicians as possible, the contact information was not completely up to date; therefore, not all clinically active physicians were accessed. Addressing the study’s external validity, we consider that our findings can apply to other primary care contexts in which general practitioners are managing comprehensive medication lists. However, the results might not be generalised to secondary care, where specialist doctors are often managing selected medications. The generalisability to primary care in other countries is also limited to similar contexts, with the Swedish one described in our study. For further validation, the questionnaire needs to be tested in other countries and contexts. Also, further studies are needed to support our findings.

### Clinical implications and future research

This study contributes new insights for future efforts to increase primary care physicians’ inclination to perform medication reconciliations in older patients with multimorbidity, with the prospect of reducing DRPs. Influencing physicians caring for multimorbid older persons is particularly important due to the frail nature of this patient category and their susceptibility to DRPs.^43^ Our findings suggest that interventions targeting attitudes and perceived behavioural control can increase physicians’ behavioural intention to perform medication reconciliation. Targeting attitudes can improve understanding of medication reconciliation’s significance and its impact on patient safety and treatment outcomes. Addressing this requires continuous education, ongoing discussions and early integration into medical training to foster behavioural change. Additionally, the survey itself could serve an interventionist role by prompting physicians to reflect on their prescribing practices. Future studies can evaluate such interventions. Furthermore, among physicians with less than 10 years of experience, it is essential that medication reconciliation is included as a part of continuing education.

## Conclusion

In this study, we developed and validated a theory-based questionnaire targeting attitudes, perceived norms, perceived behavioural control and behavioural intentions amongst primary care physicians towards performing a medication reconciliation in patients with multimorbidity. We found attitudes and perceived behavioural control to be appropriate predictors to target in future efforts to affect primary care physicians’ intention to perform a medication reconciliation in patients with multimorbidity. These findings provide important insights into how future interventions targeting behavioural predictors can be developed.

## Supplemental Material

sj-docx-1-taw-10.1177_20420986251360916 – Supplemental material for How prone are Swedish general practitioners to perform medication reconciliation? A theory-based survey study Supplemental material, sj-docx-1-taw-10.1177_20420986251360916 for How prone are Swedish general practitioners to perform medication reconciliation? A theory-based survey study by Sarah Thelin, Sara Modig and Veronica Milos Nymberg in Therapeutic Advances in Drug Safety

sj-docx-2-taw-10.1177_20420986251360916 – Supplemental material for How prone are Swedish general practitioners to perform medication reconciliation? A theory-based survey study Supplemental material, sj-docx-2-taw-10.1177_20420986251360916 for How prone are Swedish general practitioners to perform medication reconciliation? A theory-based survey study by Sarah Thelin, Sara Modig and Veronica Milos Nymberg in Therapeutic Advances in Drug Safety

sj-docx-3-taw-10.1177_20420986251360916 – Supplemental material for How prone are Swedish general practitioners to perform medication reconciliation? A theory-based survey study Supplemental material, sj-docx-3-taw-10.1177_20420986251360916 for How prone are Swedish general practitioners to perform medication reconciliation? A theory-based survey study by Sarah Thelin, Sara Modig and Veronica Milos Nymberg in Therapeutic Advances in Drug Safety

## Declarations

**Ethics approval and consent to participate:** An advisory statement was given from the Swedish Ethical Review Authority (Registry number 2023-01831-01) confirming that no ethical approval was needed and that there were no ethical objections to the study according to the Swedish Ethical Review Act (SFS 2003:460). Only anonymous data were used, and no questions on sensitive topics were asked. Before taking part in the study, the participants were provided with information on the purpose and method as well as the voluntary nature of their participation. All participants who completed the questionnaire provided informed consent to participate in the study.

**Consent for publication:** Not applicable.

**Availability of data and materials:** Data available on reasonable request from the author.
