# Supplementary material for: How prone are Swedish general practitioners to perform medication reconciliation? A theory-based survey study
Source: Ther Adv Drug Saf. 2025 Jul 25;16:20420986251360916. doi: 10.1177/20420986251360916 (PMC12304613; doi:10.1177/20420986251360916)
Supplement: sj-docx-1-taw-10.1177_20420986251360916 – Supplemental material for How prone are Swedish general practitioners to perform medication reconciliation? A theory-based survey study [file sj-docx-1-taw-10.1177_20420986251360916.docx]

Supplementary File 1: Questionnaire

**Updating the medication list in multimorbid patients in primary care**

Thank You for Your participation in this study which deals with the attitude of primary care physicians to updating the medication list in the patient record.

It will take about 5-10 minutes to answer the survey. Most of the questions are answered by ticking a number; some questions require a little more time to answer. Some questions are worded in a similar way but they are different. It is important for the study that you answer all the questions. Don't think too long about the answer because we are interested in your spontaneous thoughts and experiences. Your answers are treated confidentially.

**1. My legal sex is:** Woman Man

**2. My age is**: ≤35 36-45 46-55 56-65 ≥65 years

**3. My work experience after certification is**: 0-3 4-10 11-20 ≥21 years

**4.** **From memory, approximately how many of the last 10 multimorbid patients you met did you manage to update the medication list so that you feel confident that it is correct?**

| 0 | 1 | 2 | 3 | 4 | 5 | 6 | 7 | 8 | 9 | 10 |
| --- | --- | --- | --- | --- | --- | --- | --- | --- | --- | --- |
|  |  |  |  |  |  |  |  |  |  |  |

|  |  | ***Strongly disagree Strongly agree*** | | | | | | | |
| --- | --- | --- | --- | --- | --- | --- | --- | --- | --- |
| **5.** | **My current way of working is to update the medication list in multimorbid patients** |  | 1 | 2 | 3 | 4 | 5 | 6 | 7 |

**6.** **In general, to update the medication list:**

***Strongly disagree Strongly agree***

| 1. Reduces the risk of unnecessary prescribing | 1 | 2 | 3 | 4 | 5 | 6 | 7 |
| --- | --- | --- | --- | --- | --- | --- | --- |
| 1. Reduces the risk of admissions | 1 | 2 | 3 | 4 | 5 | 6 | 7 |
| 1. Increases patient safety | 1 | 2 | 3 | 4 | 5 | 6 | 7 |
| 1. Reduces the time required for the entire doctor's visit in the long-term | 1 | 2 | 3 | 4 | 5 | 6 | 7 |
| 1. Reduces the likelihood of drug-related problems | 1 | 2 | 3 | 4 | 5 | 6 | 7 |

**7. How important is it to…**

# **Unimportant Important**

| 1. avoid prescribing unnecessary drugs? | 1 | 2 | 3 | 4 | 5 | 6 | 7 |
| --- | --- | --- | --- | --- | --- | --- | --- |
| 1. reduce the risk of drug-related hospitalisations? | 1 | 2 | 3 | 4 | 5 | 6 | 7 |
| 1. reduce the likelihood that the patient will seek treatment again due to a drug-related problem? | 1 | 2 | 3 | 4 | 5 | 6 | 7 |
| 1. have enough time for medication reconciliation during the patient’s visit? | 1 | 2 | 3 | 4 | 5 | 6 | 7 |
| 1. reduce the patient's risk of drug-related problems in the future? | 1 | 2 | 3 | 4 | 5 | 6 | 7 |

**8.** **If I routinely manage multimorbid patients by updating the drug list, my life as a general practitioner will generally be easier in the long run**

***Strongly disagree Strongly agree***

| 1 | 2 | 3 | 4 | 5 | 6 | 7 |
| --- | --- | --- | --- | --- | --- | --- |
|  |  |  |  |  |  |  |

**9. Treating multimorbid patients by updating the medication list is...**

***Strongly disagree Strongly agree***

| 1. usually a better treatment option | 1 | 2 | 3 | 4 | 5 | 6 | 7 |
| --- | --- | --- | --- | --- | --- | --- | --- |
| 1. satisfactory more often than unsatisfactory | 1 | 2 | 3 | 4 | 5 | 6 | 7 |

**10. About updating the medication list:  *Strongly disagree Strongly agree***

| 1. Many people who are important to me (colleagues, patients, the manager) think that I should update drug lists for multimorbid patients | 1 | 2 | 3 | 4 | 5 | 6 | 7 |
| --- | --- | --- | --- | --- | --- | --- | --- |
| 1. I am expected to update drug lists for multimorbid patients | 1 | 2 | 3 | 4 | 5 | 6 | 7 |
|  |  |  |  |  |  |  |  |

**11. When it comes to updating a patient's medication list, how motivated are you to do what...**

***Not motivated at all Fully motivated***

| 1. the primary care colleagues think you should | 1 | 2 | 3 | 4 | 5 | 6 | 7 |
| --- | --- | --- | --- | --- | --- | --- | --- |
| 1. the inpatient colleagues think you should | 1 | 2 | 3 | 4 | 5 | 6 | 7 |
| 1. the manager thinks you should | 1 | 2 | 3 | 4 | 5 | 6 | 7 |

**12. How confident are you in your ability/competence**

***Not confident at all Very confident***

| 1. to update the medication list at each visit for multimorbid patients? | 1 | 2 | 3 | 4 | 5 | 6 | 7 |
| --- | --- | --- | --- | --- | --- | --- | --- |
| 1. to end a visit for a multimorbid patient whom you have treated by handing out the medication list? | 1 | 2 | 3 | 4 | 5 | 6 | 7 |
|  |  |  |  |  |  |  |  |

# **13. With the current working methods and conditions, how confident are you in your own ability to update the medication list in patients with multimorbidity who**

***Not confident at all Very confident***

| 1. come on scheduled check? | 1 | 2 | 3 | 4 | 5 | 6 | 7 |
| --- | --- | --- | --- | --- | --- | --- | --- |
| 1. have multiple prescribers? | 1 | 2 | 3 | 4 | 5 | 6 | 7 |
| 1. use many medications? | 1 | 2 | 3 | 4 | 5 | 6 | 7 |

**14. Based on the information you have at the visit:**

***Strongly disagree Strongly agree***

| 1. I want and can update the drug list in patients with multimorbidity | 1 | 2 | 3 | 4 | 5 | 6 | 7 |
| --- | --- | --- | --- | --- | --- | --- | --- |
| 1. I am convinced that I can update the medication list in multimorbid patients even with several prescribers | 1 | 2 | 3 | 4 | 5 | 6 | 7 |
| 1. I can overcome all obstacles (e.g. lack of time), to update the medication list in patients with multimorbidity | 1 | 2 | 3 | 4 | 5 | 6 | 7 |

**15. Management of patients with multimorbidity.**

***Strongly disagree Strongly agree***

| 1. When managing multi-morbid patients, I automatically plan to update their drug list. | 1 | 2 | 3 | 4 | 5 | 6 | 7 |
| --- | --- | --- | --- | --- | --- | --- | --- |
| 1. I want to treat multimorbid patients by updating their medication list. | 1 | 2 | 3 | 4 | 5 | 6 | 7 |
| 1. I strive to manage multimorbid patients by updating their medication list. | 1 | 2 | 3 | 4 | 5 | 6 | 7 |
