# Supplementary material for: How prone are Swedish general practitioners to perform medication reconciliation? A theory-based survey study
Source: Ther Adv Drug Saf. 2025 Jul 25;16:20420986251360916. doi: 10.1177/20420986251360916 (PMC12304613; doi:10.1177/20420986251360916)
Supplement: sj-docx-2-taw-10.1177_20420986251360916 – Supplemental material for How prone are Swedish general practitioners to perform medication reconciliation? A theory-based survey study [file sj-docx-2-taw-10.1177_20420986251360916.docx]

Supplementary File 2: CHERRIES Checklist

| **ITEM CATEGORY** | **CHECK-LIST ITEM** | **EXPLANATION** |
| --- | --- | --- |
| **Design** | Describe survey design | Web-based survey to primary care physicians in southern Sweden. |
| **IRB (Institutional Review Board) approval and informed consent process** | Ethical approval | The Swedish Ethical Review Authority assessed that no ethical approval was needed, instead an advisory remark was obtained (Registry number 2023-01831-01). |
|  | Informed consent | In the invitation e-mail, the purpose of the study, the length of time the survey would take, contact information to the principal investigator for questions, and the anonymous nature of their participation was informed. The e-mail included a link to the survey. Upon answering the survey, their response was deidentified and coded automatic by the software. |
|  | Data protection | Data was collected through the secure electronic data-base manager Sunet Survey. All answers were anonymized. |
| **Development and pre-testing** | Development and testing | Construct validity and readability of the survey in paper format was assessed by two physicians to ensure relevance of items and wordings and led to minor changes in wording and formatting. Then, we performed a pilot study at three primary health care centres by distributing the questionnaire to 20 primary care physicians. For each of the primary outcome variables (attitudes, perceived norms, perceived behavioural control, and behavioural intention) the mean of the item scores was calculated to give an overall score. Missing values were replaced with the mean value for the item (imputation). Internal consistency was measured with Cronbach’s α coefficient (CA) with an accepted value >0.6. For items with low CA subitems were removed to improve CA. A lower CA was noted amongst questions with an inverted scale resulting in alterations to ensure a unanimous scale throughout the survey. Temporal stability of the questionnaire was assessed by redistributing the questionnaire to the same 20 respondents after two weeks. For identifying individual respondents whilst they were remaining anonymous, they were asked to mark their survey sheets with a personal code or figure. The Pearson correlation coefficient was calculated to examine test-retest reliability. This led us to change the structure of some questions to improve the final questionnaire. |
| **Recruitment process and description of the sample having access to the survey** | Open survey vs closed survey | Closed survey. |
|  | Contact mode | Participants were invited to the survey by an individual link to their e-mail. Upon answering the survey, their response was deidentified and coded automatic by the software. |
|  | Advertising the survey | The invited participants received an e-mail with information on the survey along with the link to attend. Two additional e-mails were sent to non-responders. |
| **Survey administration** | Web/E-mail | Web-based survey. |
|  | Context | Not applicable. |
|  | Mandatory/voluntary | Not applicable. |
|  | Incentives | No incentives were offered those who choose to participate in the survey. |
|  | Time/Date | The survey was open between the 5^th^ of February to the 28^th^ of March 2024. |
|  | Randomization of items or questionnaires | No randomization was applied. |
|  | Adaptive questioning | All surveys had the same questions. |
|  | Number of Items | 16 main questions with in total 34 questions, all subitems included. |
|  | Number of screens (pages) | All questions could be viewed in the same page. |
|  | Completeness check | All items were mandatory with the selection of one response. A non-response option was not provided. |
|  | Review step | All responses could be seen at the same page, consequently, no Back button was necessary. No additional Review step before submitting was provided. |
| **Response rates** | Unique site visitor | Unique site visitors were not estimated. |
|  | View rate | View rates were not estimated. |
|  | Participation rate | Participation rates were not estimated. |
|  | Completion rate | Completions rates were not estimated. |
| **Preventing multiple entries from the same individual** | Cookies, IP-check, log-file analysis, or registration | Multiple entries were prevented through registration, namely, each invited participant received an individual survey-link. The respondents were not able to change their answers after the survey was sent in. Reminders were only sent to respondents that had not already answered the survey. Upon answering the survey, the participants responses were deidentified and coded automatic by the software. |
| **Analysis** | Handling of incomplete questionnaires | Not applicable, only completed sent in surveys were analysed and all questions were mandatory. |
|  | Questionnaires submitted with an atypical timestamp | A cut-off point for the time needed to complete the survey was not used. |
|  | Statistical correction | No correctional analysis was made. |
