# Supplementary material for: How prone are Swedish general practitioners to perform medication reconciliation? A theory-based survey study
Source: Ther Adv Drug Saf. 2025 Jul 25;16:20420986251360916. doi: 10.1177/20420986251360916 (PMC12304613; doi:10.1177/20420986251360916)
Supplement: sj-docx-3-taw-10.1177_20420986251360916 – Supplemental material for How prone are Swedish general practitioners to perform medication reconciliation? A theory-based survey study [file sj-docx-3-taw-10.1177_20420986251360916.docx]

Distribution of scores for individual items on a 7-point Likert scale*

| **Predictor** | **Item** | | **Mean (SD)** | **Median (IQR)**** |
| --- | --- | --- | --- | --- |
| Attitudes | In general, to update the medication list: | a) Reduces the risk of unnecessary prescribing | 6.45 (1.107) | 7 (6-7-7) |
|  |  | b) Reduces the risk of admissions | 5.66 (1.446) | 6 (5-6-7) |
|  |  | c) Increases patient safety | 6.75 (0.787) | 7 (7-7-7) |
|  |  | d) Reduces the time required for the entire doctor's visit in the long-term | 5.91 (1.505) | 7 (5-7-7) |
|  |  | e) Reduces the likelihood of drug-related problems | 6.49 (0.956) | 7 (6-7-7) |
|  | How important is it to… | a) avoid prescribing unnecessary drugs? | 6.54 (0.903) | 7 (6-7-7) |
|  |  | b) reduce the risk of drug-related hospitalisations? | 6.81 (0.599) | 7 (7-7-7) |
|  |  | c) reduce the likelihood that the patient will seek treatment again due to a drug-related problem? | 6.62 (0.755) | 7 (6-7-7) |
|  |  | d) have enough time for medication reconciliation during the patient’s visit? | 6.64 (0.831) | 7 (7-7-7) |
|  |  | e) reduce the patient's risk of drug-related problems in the future? | 6.74 (0.565) | 7 (7-7-7) |
|  | If I routinely manage multimorbid patients by updating the medication list, my life as a general practitioner will generally be easier in the long run | | 6.32 (1.153) | 7 (6-7-7) |
|  | Treating multimorbid patients by updating the medication list is... | a) usually a better treatment option | 6.41 (0.997) | 7 (6-7-7) |
|  |  | b) satisfactory more often than unsatisfactory | 6.16 (1.185) | 7 (6-7-7) |
| *Attitudes overall score:* | | | *6.42 (0.623)* | *6.54 (6.21-6.54-6.85)* |
| Subjective norms | About updating the medication list: | a) Many people who are important to me (colleagues, patients, the manager) think that I should update medication lists for multimorbid patients | 5.50 (1.658) | 6 (5-6-7) |
|  |  | b) I am expected to update medication lists for multimorbid patients | 5.93 (1.413) | 7 (5-7-7) |
|  | When it comes to updating a patient's medication list, how motivated are you to do what... | a) the primary care colleagues think you should | 5.81 (1.424) | 6 (5-6-7) |
|  |  | b) the inpatient colleagues think you should | 4.87 (1.810) | 5 (4-5-6) |
|  |  | c) the manager thinks you should | 5.13 (1.765) | 5 (4-5-7) |
| *Subjective norms overall score:* | | | *5.45 (1.221)* | *5.60 (4.60-5.60-6.40)* |
| Perceived behavioural control | How confident are you in your ability/competence | a) to update the medication list at each visit for multimorbid patients? | 5.79 (1.068) | 6 (5-6-7) |
|  |  | b) to end a visit for a multimorbid patient whom you have treated by handing out the medication list? | 5.93 (1.160) | 6 (5-6-7) |
|  | With the current working methods and conditions, how confident are you in your own ability to update the medication list in patients with multimorbidity who | a) come on scheduled check? | 5.89 (1.197) | 6 (5-6-7) |
|  |  | b) have multiple prescribers? | 4.36 (1.494) | 4 (4-4-5) |
|  |  | c) use many medications? | 5.20 (1.302) | 5 (4-5-6) |
|  | Based on the information you have at the visit: | a) I want and can update the medication list in patients with multimorbidity. | 5.68 (1.304) | 6 (5-6-7) |
|  |  | b) I am convinced that I can update the medication list in multimorbid patients even with several prescribers | 4.71 (1.572) | 5 (4-5-6) |
|  |  | c) I can overcome all obstacles (e.g. lack of time), to update the medication list in patients with multimorbidity | 3.64 (1.171) | 4 (2-4-5) |
| *Perceived behavioural control overall score:* | | | *5.15 (0.994)* | *5.25 (4.50-5.25-5.88)* |
| Generalised intention | Management of patients with multimorbidity. | a) When managing multi-morbid patients, I automatically plan to update their medication list. | 5.65 (1.465) | 6 (5-6-7) |
|  |  | b) I want to treat multimorbid patients by updating their medication list. | 6.45 (1.005) | 7 (6-7-7) |
|  |  | c) I strive to manage multimorbid patients by updating their medication list. | 6.40 (0.982) | 7 (6-7-7) |
| *Generalised intention overall score:* | | | *6.17 (0.977)* | *6.33 (5.67-6.33-7.00)* |

*1=Strongly disagree, 7=Strongly agree
**IQR=Interquartile range
